# Ecological and Human Health Risk Assessment of Heavy Metals in Mining-Affected River Sediments in the Peruvian Central Highlands

**DOI:** 10.3390/toxics13090783

**Published:** 2025-09-16

**Authors:** María Custodio, Samuel Pizarro, Javier Huarcaya, Kevin Ortega, Dennis Ccopi

**Affiliations:** 1 Environmental Science & Health Research Group, Facultad de Medicina Humana, Universidad Nacional del Centro del Perú, Av. Mariscal Castilla N° 3909, Huancayo 12000, Junin, Peru; mcustodio@uncp.edu.pe (M.C.); javierdark01@gmail.com (J.H.); 2 Dirección de Servicios Estratégicos Agrarios, Estación Experimental Agraria Santa Ana, Instituto Nacional de Innovación Agraria (INIA), Carretera Saños Grande–Hualahoyo Km 8 Santa Ana, Huancayo 12000, Junin, Peru; samuel.pizarro@untrm.edu.pe; 3 Faculty of Engineering, Professional School of Environmental Engineering, Universidad Continental, Huancayo 12000, Junin, Peru; kevinorqu@gmail.com; 4 Dirección de Recursos Genéticos y Biotecnología, Estación Experimental Agraria Santa Ana, Instituto Nacional de Innovación Agraria (INIA), Carretera Saños Grande–Hualahoyo Km 8 Santa Ana, Huancayo 12000, Junin, Peru

**Keywords:** heavy metal, ecological risk, human health risk, river sediments, mining, toxicity, water pollution

## Abstract

Heavy metal contamination in rivers is a serious environmental and public health concern, especially in areas affected by mining. This study evaluated the levels of contamination and the associated ecological and carcinogenic risks in the sediments of the Cunas River, located in the central highlands of Peru. Sediment samples were collected from upstream and downstream sections. Several metals and metalloids were analyzed, including copper (Cu), chromium (Cr), iron (Fe), manganese (Mn), molybdenum (Mo), nickel (Ni), lead (Pb), vanadium (V), zinc (Zn), antimony (Sb), arsenic (As), and cadmium (Cd). The ecological risk assessment focused on ten of these elements, while carcinogenic and non-carcinogenic risks were assessed for seven metals selected based on their toxicological importance. The results showed that Cd and Pb concentrations were higher in the downstream section. Cd and As exceeded ecological risk thresholds. Regarding human health, As and Pb surpassed the acceptable limits for both the Hazard Index (HI) and the Potential Carcinogenic Risk (PCR). According to EPA guidelines, these values indicate a potentially significant lifetime cancer risk. The main exposure routes include direct contact with sediments and the consumption of aquatic organisms. Continuous monitoring, phytoremediation actions, and restrictions on the use of contaminated water are strongly recommended to reduce ecological and health risks.

## 1. Introduction

Mining activities constitute one of the primary anthropogenic sources of heavy metal contamination in terrestrial and aquatic ecosystems worldwide [1]. The extraction and processing of mineral resources release substantial quantities of potentially toxic elements, including arsenic (As), cadmium (Cd), lead (Pb), mercury (Hg), copper (Cu), chromium (Cr), iron (Fe), manganese (Mn), molybdenum (Mo), nickel (Ni), vanadium (V), zinc (Zn), and antimony (Sb), into the environment [2]. These contaminants progressively accumulate in soils and water bodies, leading to biodiversity loss and ecosystem dysfunction. The persistence of heavy metal contamination is particularly concerning due to the non-biodegradable nature of these elements and is often exacerbated by inadequate mining waste management practices and the intensification of extractive activities in ecologically sensitive regions [3].

The bioaccumulation potential and chronic toxicity of heavy metals pose significant threats to human health through multiple exposure pathways. Epidemiological studies have established associations between heavy metal exposure and various adverse health outcomes, including respiratory diseases, renal dysfunction, neurological disorders, and multiple cancer types [4]. At the molecular level in cells, heavy metals increase oxidative stress through the activation of transcription factors such as NF-κB and AP-1, and through the generation of reactive oxygen species via Fenton-type reactions [5,6]. These pathogenic mechanisms have been implicated in the development of pulmonary [7], hepatic [8], and renal carcinomas [9].

In aquatic environments, sediments serve as both primary sinks and long-term reservoirs for heavy metals [10]. However, changes in physicochemical parameters including pH, salinity, organic matter content, and redox potential can promote the remobilization of sequestered metals into the overlying water column, thereby reactivating their bioavailability and toxicity [11,12,13]. This dynamic process not only prolongs ecological exposure but also amplifies impacts on aquatic biota and facilitates contaminant transfer through trophic webs. Consequently, sediment quality assessment represents a critical component in evaluating ecosystem health and environmental risk in riverine systems [10,14].

Heavy metal contamination of river sediments represents an escalating environmental challenge in Peru, particularly within Andean watersheds subjected to intensive mining operations. The Cunas River micro-watershed, situated in Peru’s central Andes, exemplifies this regional problem [15,16]. Continuous discharge of mining effluents has substantially degraded water quality and compromised the ecological integrity of aquatic ecosystems throughout this watershed [17,18,19]. Contaminants originating from mine tailings and metallurgical processes have demonstrably impacted local flora and fauna while creating human health risks through bioaccumulation in trophic webs [20,21].

Given the persistence and toxicity of heavy metals, comprehensive risk assessment requires integrated methodological approaches capable of evaluating both ecological impacts and human health implications. Various contamination indices and predictive models have been developed to quantify contaminant accumulation in sediments and forecast potential effects on aquatic ecosystems and exposed [11]. Research conducted in mining-impacted regions has demonstrated that elements such as As, Cd, and Pb present substantial human health risks due to their bioaccumulative properties and carcinogenic potential. The co-occurrence of multiple metals in sediment matrices further compounds ecological risk by disrupting both biotic and abiotic components of aquatic systems [3,14,22].

This study evaluates the ecological and human health risks associated with heavy metal contamination in sediments of the Cunas River, central Peruvian Andes. Ecological risk assessment encompassed twelve metals: copper (Cu), chromium (Cr), iron (Fe), manganese (Mn), molybdenum (Mo), nickel (Ni), lead (Pb), vanadium (V), zinc (Zn), antimony (Sb), arsenic (As), and cadmium (Cd). Human health risk assessment focused on five priority metals—arsenic (As), cadmium (Cd), nickel (Ni), chromium (Cr), and lead (Pb)—using age-specific exposure models. The assessment encompassed communities located in both upper and lower reaches of the Cunas River sub-basin. This research integrates spatial analysis, toxicological modeling, and multiple environmental indicators to identify critical zones of environmental and public health concern. The findings will provide evidence-based recommendations for developing effective mitigation strategies and sustainable watershed management practices.

## 2. Materials and Methods

### 2.1. Description of the Study Area

The Cunas River watershed is situated in the Junín region of the central Peruvian Andes, originating at an elevation of 5180 m above sea level within the Andean cordillera and encompassing a drainage area of 1845 km^2^. The watershed extends between 11°45′–12°20′ S latitude and 75°15′–75°45′ W longitude, traversing Concepción [23].

**Figure 1:**
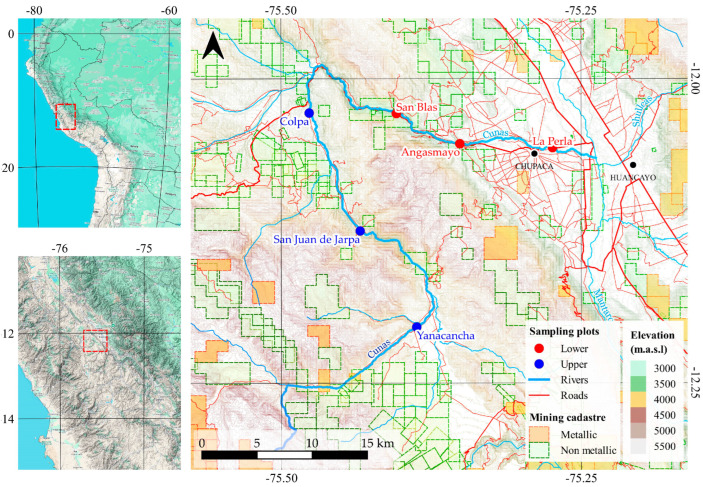
Map of the study area with sampling plots categorized by elevation: red dots represent lower elevation plots, and blue dots represent upper elevation plots.

The river system (Figure 1) is subject to significant anthropogenic pressure from mining operations, with polymetallic extraction activities concentrated primarily in the upper watershed areas. Heavy metal contamination enters the sedimentary environment through multiple pathways, including acid mine drainage, surface runoff, and erosion of mining waste deposits, resulting in progressive environmental degradation [24].

Six sampling stations were strategically established along the longitudinal profile of the Cunas River to evaluate the spatial distribution of ecological and carcinogenic risks associated with mining-derived heavy metals. The sampling design incorporated two distinct zones—the upper and lower river reaches—to facilitate assessment of altitudinal gradients in contamination patterns.

The upper river zone, situated at elevations ranging from 3800 to 4300 m.a.s.l., encompasses the communities of Yanacancha, San Juan de Jarpa, and Colpa. The lower river zone, located at elevations between 3200 and 3500 m.a.s.l., includes the communities of Angasmayo, San Blas, and La Perla. This zonation scheme was established based on the altitudinal distribution of human settlements along the river corridor, enabling systematic analysis of spatial variability in heavy metal accumulation and associated ecological and health risks across distinct segments of the fluvial system.

### 2.2. Fluvial Sediment Sampling

Sediment sampling was conducted during the wet season (February and March) to capture peak contaminant loading conditions. Six sampling stations were established along the Cunas River, with four discrete collection points designated at each station to ensure spatial representativeness within each reach. Sediment samples were collected from the upper 10 cm of the riverbed using a stainless-steel corer specifically designed for aquatic sediment extraction.

To minimize spatial heterogeneity and obtain representative samples, the four subsamples collected at each station were homogenized to create 500 g composite samples. All samples were immediately placed in pre-sterilized, airtight polyethylene containers and maintained under refrigerated conditions (4 °C) during transport to the analytical laboratory to preserve sample integrity and prevent contamination or degradation of target analytes.

### 2.3. Heavy Metal Determination, Quality Control, and Assurance

Heavy metal analysis was performed following standardized environmental quality protocols validated by the Peruvian National Institute of Quality (INACAL). Sample preparation involved complete acid digestion using a tri-acid mixture of concentrated hydrofluoric acid (HF), nitric acid (HNO_3_), and perchloric acid (HClO_4_) in a 5:2:1 volumetric ratio. Metal quantification was conducted using inductively coupled plasma mass spectrometry (ICP-MS) on a PerkinElmer NexION^®^ 1000 instrument (Waltham, MA, USA).

Quality assurance and quality control (QA/QC) protocols were implemented throughout the analytical process to ensure data reliability and accuracy. Quality control measures included (i) triplicate analysis of all samples to assess analytical precision, (ii) procedural blanks processed identically to the samples to monitor contamination, (iii) certified reference materials (CRMs) analyzed for each target metal to verify accuracy, and (iv) instrumental precision evaluation through replicate measurements [25]. Recovery rates for certified reference materials were within acceptable limits for all analyzed metals, confirming the validity of the analytical methodology. These rigorous quality control procedures ensured the production of reliable analytical results.

Method limits of quantification (LOQs) were calculated from the instrumental LOQs of the PerkinElmer NexION^®^ 1000 ICP–MS, considering the digestion of 0.50 g of sediment in 50 mL. The LOQs were 0.01 mg/kg for As, Cd, Cr, Cu, Mn, and Ni; 0.02 mg/kg for Pb and Sb; 0.05 mg/kg for Mo; 0.10 mg/kg for Zn; and 0.50 mg/kg for Fe.

### 2.4. Risk Assessment Methods

To assess sediment contamination and ecological risk, seven quantitative indices were applied to twelve elements (Cu, Cr, Fe, Mn, Mo, Ni, Pb, V, Zn, Sb, As, and Cd). Background values were primarily based on average concentrations in the upper continental crust proposed by Taylor & Mclennan (1995) [26] and Turekian & Wedepohl [27]. For ecological risk estimation, toxic response factors established by Hakanson (1980) [28] were also incorporated. All formulas used for the calculation of these indices are provided in Table A1.

The Contamination Factor (Cf) was calculated as the ratio between the concentration of each metal in sediments and its corresponding background value. This index allows classification of contamination levels from low to extremely high [29]. Based on Cf values, the Pollution Load Index (PLI) [30,31], the Modified Contamination Degree (mCd) [32], and the Geoaccumulation Index (Igeo) [33,34] were derived. The latter incorporates a correction factor of 1.5 to account for natural variability and classifies contamination into seven levels, ranging from unpolluted to extremely polluted.

The Enrichment Factor (EF) was used to identify the potential anthropogenic origin of the metals, with iron (Fe) selected as the reference element due to its geochemical stability. EF values greater than 1.5 indicate anthropogenic enrichment [30,31].

#### 2.4.1. Potential Ecological Risk (RI)

The RI index is calculated by summing the ecological risk factors (Er) corresponding to the heavy metals present in the sediments [31]. This index allows for the assessment of the overall ecological risk level posed by heavy metal concentrations in the studied samples. It is determined using Equation (1), which incorporates the biological toxic factor (T) of each metal.

(1) $$E_{r}^{i}=T_{f}^{i}\times {CF}_{r}^{i};Ri=\sum_{i=n}^{n}E_{r}^{i}$$

where $E_{r}^{i}$ represents the individual risk index, $T_{f}^{i}$ is the toxicity factor, ${CF}_{r}^{i}$ is the Contamination Factor, and Ri is the total risk index [35]. According to its value, the RI index classifies ecological risk into four levels: low (Ri ≤ 95), moderate (95 < R Ri ≤ 190), considerable (190 < Ri ≤ 380), and very high (Ri > 380).

#### 2.4.2. Site Ranking Index (SRI)

The Site Ranking Index (***SRI***) assesses contamination levels in a sampling area by considering metal concentrations across different matrices, eliminating arbitrary or subjective classifications [36,37]. It is calculated using Equation (2).

(2) $$W=\frac{\sum n_{i}}{\sum i};SRI=\frac{W}{S}\times 100$$

where W is the weighted contamination index, S is the number of evaluated contaminants, n is the contamination ranking in ascending order (Cf and Igeo), i is the position of the metal, and *SRI* is the contamination risk index. The *SRI* categories are divided into four levels—low contamination, when *SRI* < median − SD; moderate contamination, if median − SD < *SRI* < median; high contamination, when median < *SRI* < median + SD; and severe contamination, if *SRI* > median + SD—according to Zhang et al. (2020) [36].

### 2.5. Risk to Human Health: Carcinogenic and Non-Carcinogenic Risk Assessment Methods

Carcinogenic and non-carcinogenic risks associated with human exposure to heavy metals in the watershed were assessed following the EPA-USA model. The analysis considered three main exposure pathways: ingestion, inhalation, and dermal contact [38,39]. To calculate these risks, the Chronic Daily Dose (CDD, mg/kg/day) equation was used, as shown in Equations (3)–(5), evaluating the impact of each exposure route to the contaminants.

(3) $${CDD}_{ingestion}=\frac{CM\times IR\times ED\times EF}{ABW\times AET}\times Cf$$

(4) $${CDD}_{inhalation}=\frac{CM\times IHR\times ED\times EF}{ABW\times AET\times PEF}$$

(5) $${CDD}_{dermal}=\frac{CM\times SA\times SAF\times DAF\times ED\times EF}{ABW\times AET}\times Cf$$

For the calculation of CDD (Chronic Daily Dose), two distinct age groups were considered: children and adults. Each group presents significant differences in exposure parameters, such as average body weight (ABW), sediment ingestion rate (IR), inhalation rate (IHR), dermal contact surface area (SA), exposure duration (ED), and exposure frequency (EF), which were adjusted according to EPA (Environmental Protection Agency) reference values.

The inclusion of these two groups allows for a more precise assessment of the risk associated with heavy metal exposure in sediments, considering physiological variability and behavioral patterns that influence the absorption and accumulation of contaminants at each stage of human development.

Non-carcinogenic risk was assessed using the HQ index in Equation (6), calculated as the ratio between the Chronic Daily Dose (CDD) and the Reference Dose (RfD) for each metal and exposure route (ingestion, inhalation, and dermal). These values were integrated into the HI, shown in Equation (7), where HI < 1 indicates an insignificant risk, and HI > 1 suggests a possible chronic adverse effect [40].

On the other hand, carcinogenic risk was determined through the CR index, calculated using Equation (8), incorporating CDD and specific factors for each exposure route, including the slope factor (SF), inhalation unit risk (IUR), and gastrointestinal absorption factor (G). The sum of the CR values generated the TCR index, Equation (9), which is interpreted as unacceptable risk if TCR > 1.00 × 10^−4^, acceptable risk if 1.00 × 10^−6^ < TCR < 1.00 × 10^−4^, and no significant risk if TCR < 1.00 × 10^−6^. The values of RfD, SF, IUR, and G were obtained from Saber et al. (2024) [41].

(6) $${HQ}_{(ingestion/inhalacion)/dermico}=\frac{{CDD}_{ind/inh/der}}{{RfD}_{ind/inh/der}}$$

(7) $${HI=\sum HQ}_{ind/inh/der}$$

(8) $${CR}_{ing}={CDD}_{ing}\times Sf;{CR}_{inh}={CDD}_{inh}\times IUR;{CR}_{der}={CDD}_{der}$$

(9) $$TCR{=\sum CR}_{ing/inh/der}$$

### 2.6. Statical Analysis

The statistical analysis was performed using R software (version 4.4.3, San Francisco, CA, USA), employing various techniques to assess distribution and sociological impact. Graphical representations were generated using the heatmap function [42] to visualize the correlation between metals and detect association patterns in their concentrations through the FactoMineR package (version 2.12, Rennes, France) [43]. Additionally, a cluster analysis was applied using hierarchical clustering methods to identify areas with similar environmental characteristics.

A Principal Component Analysis (PCA) was also conducted to reduce data dimensionality and determine the variables that explain the greatest variability in metal distribution, utilizing the dendextend package [44]. Finally, the Wilcoxon rank sum exact test was performed to evaluate significant differences in metal concentrations between study areas or groups.

## 3. Results

### 3.1. Heavy Metal Analysis in Sediment

Heavy metal concentrations in Cunas River sediments exhibited distinct spatial patterns, with significantly higher levels observed in the lower zone compared to the upper zone (Table 1 and Table A2). The most abundant elements in the sediments were Zn, V, Ni, Cu, Pb, and As, demonstrating pronounced accumulation in downstream reaches. Arsenic and cadmium concentrations exceeded upper continental crust (UCC) reference values of 1.5 mg/kg and 0.1 mg/kg, respectively. In the lower zone, As reached mean concentrations of 15.4 mg/kg (10-fold higher than UCC) and Cd attained 0.4 mg/kg (4-fold higher than UCC), while the upper zone also showed elevated levels with As at 7.7 mg/kg and Cd at 0.2 mg/kg.

Arsenic concentrations in the lower zone not only exceeded UCC benchmarks but also surpassed typical background levels, indicating significant anthropogenic enrichment. Lead concentrations notably increased from the upper zone (11.1 mg/kg) to the lower zone (23.1 mg/kg), remaining above UCC values (20 mg/kg) but below the Threshold Effect Level (TEL, 30.2 mg/kg). Similarly, zinc concentrations demonstrated a marked increase downstream, rising from 35.9 mg/kg in the upper zone to 101.3 mg/kg in the lower zone, exceeding the UCC reference value (95 mg/kg) but remaining below the TEL (124 mg/kg). Nickel concentrations also showed spatial differentiation, with mean values increasing from 7.1 mg/kg in the upper zone to 11.0 mg/kg in the lower zone, though remaining well below UCC levels (68 mg/kg).

Copper exhibited moderate spatial variation, with concentrations increasing from 6.2 mg/kg in the upper zone to 8.1 mg/kg in the lower zone, both values remaining substantially below UCC (45 mg/kg) and TEL (18.7 mg/kg) thresholds. Mercury concentrations were relatively low across both zones, with mean values of 0.2 mg/kg in the upper zone and 0.1 mg/kg in the lower zone, remaining below both UCC (0.4 mg/kg) and TEL (0.13 mg/kg) reference levels. Antimony concentrations increased downstream from 0.4 mg/kg to 1.1 mg/kg, exceeding UCC values (0.2 mg/kg) but showing moderate enrichment levels. Chromium, vanadium, iron, manganese, and molybdenum exhibited relatively stable distributions throughout the river system, with concentrations consistently remaining below their respective UCC reference values, indicating minimal anthropogenic influence for these elements.

Table A2 presents the differences in heavy metal concentrations between the lower (N = 6) and upper (N = 6) groups, revealing a differentiated impact of Sb, Cd, Mg, Mo, Ag, and Zn concentrations (*p* < 0.05), suggesting greater metal mobility and accumulation in the lower zone.

### 3.2. Correlation and Cluster Analysis of Heavy Metals in Sediments

**Figure 2:**
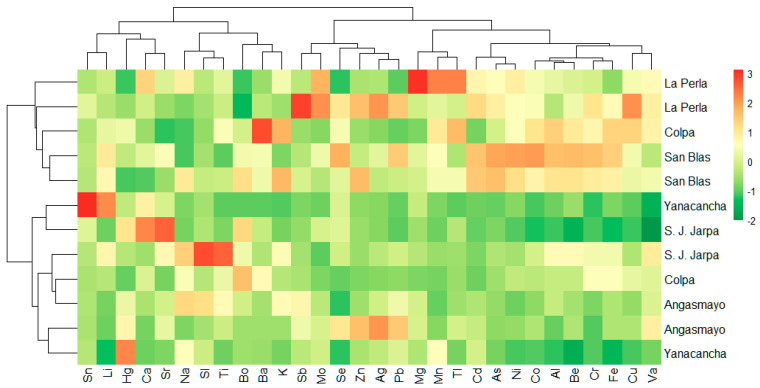
Cluster or hierarchical grouping of heavy metals with similar distribution patterns in river sediment.

Figure 2 presents a hierarchical cluster analysis heatmap of standardized heavy metal concentrations across sampling sites in the Cunas River watershed. The color-coded matrix employs a gradient scale where red indicates elevated concentrations, yellow represents intermediate levels, and green denotes lower concentrations relative to the dataset mean.

The spatial distribution analysis reveals distinct contamination patterns, with downstream sampling sites (La Perla, San Blas, and Angasmayo) consistently exhibiting higher metal concentrations, particularly for Zn, Pb, Cd, Cu, and As, as evidenced by the predominant red and orange coloration. Conversely, upstream locations (Yanacancha, San Juan de Jarpa, and Colpa) demonstrate relatively lower contamination levels, characterized by green and yellow hues across most elements. This downstream accumulation pattern suggests progressive metal loading along the river continuum, consistent with point-source contamination from mining activities.

The hierarchical clustering algorithm identifies two primary site clusters: an upstream cluster characterized by lower overall metal concentrations and a downstream cluster exhibiting elevated contamination levels. Similarly, element clustering reveals associations between metals with comparable distribution patterns, suggesting common sources or geochemical behaviors.

Notable element groupings include the clustering of toxic metals (As, Cd, Pb) and transition metals (Cu, Zn, Ni), indicating potential co-occurrence from mining-related inputs. This clustering pattern supports the hypothesis of anthropogenic contamination, with mining activities serving as the primary source of metal enrichment in the lower watershed reaches.

### 3.3. Correlation and Cluster Analysis of Heavy Metals in Sediments

For ecological risk assessment, reference values from Taylor & Mclennan (1995) [26] and Turekian & Wedepohl (1961) [27] were used to establish geochemical baselines for evaluating sediment contamination and anthropogenic enrichment.

**Figure 3:**
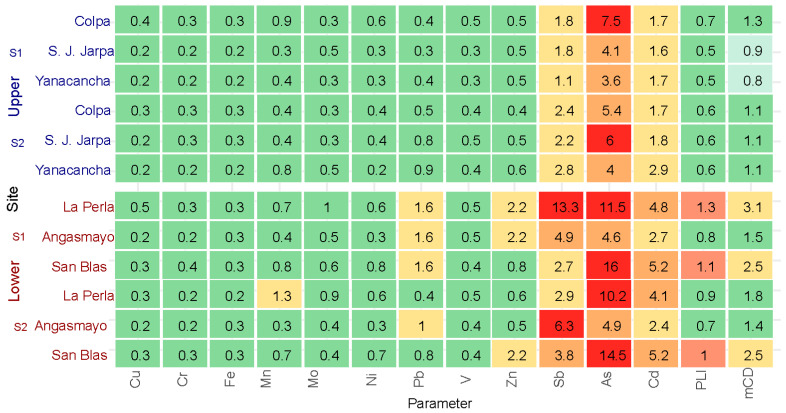
Contamination Factors by heavy metal (Cf), Pollution Load Index (PLI), and Modified Contamination Degree (mCd) of Taylor & Mclennan (1995) [26] and (Turekian & Wedepohl (1961) [27].

Figure 3 presents a heatmap of Contamination Factor (Cf) values for 12 elements, along with Pollution Load Index (PLI) and Modified Contamination Degree (mCd) across three sampling sites in two distinct zones. The color gradient indicates extreme values for Contamination Factor, with green representing low contamination (Cf < 1), yellow indicating moderate levels (Cf 1–3), orange showing high contamination (Cf 3–6), and red denoting very high contamination (Cf > 6).

The upper zone sites (Colpa, San Juan de Jarpa, and Yanacancha) exhibited predominantly green coloration, with Cf values below 1 for most elements, indicating minimal contamination controlled by natural geogenic processes. Notable exceptions include arsenic, with the highest value of As = 7.5 in Colpa (S1), and antimony, reaching Sb = 6.0 in San Juan de Jarpa (S2), both appearing as red cells indicating localized but significant enrichment.

The lower zone (La Perla, Angasmayo, and San Blas) displayed markedly elevated contamination levels, characterized by extensive red and orange coloration. Arsenic and antimony demonstrated the most severe contamination, with As reaching 16.0 in San Blas and 11.5 in La Perla, while Sb peaked at 13.3 in La Perla. Additional metals including cadmium (Cd = 5.2 in San Blas), zinc, and lead also exhibited substantial enrichment, particularly in La Perla, reflecting significant mining-related inputs.

In the upper zone, elevated Contamination Factors were recorded for As at S1 (Cf = 7.5, February) and Sb at S2 (Cf = 5.4, March). These peaks coincided with wet season sampling, when increased river discharge and runoff from upstream mining areas can enhance the transport and deposition of these elements at specific locations, a pattern also reported in other mining-impacted Andean basins [30].

The integrative contamination indices (PLI and mCd) displayed in the rightmost columns corroborated these spatial patterns. Upper zone sites maintained PLI and mCd values below 1.5 (shown in green), while lower zone locations, particularly La Perla and San Blas, exceeded critical thresholds with mCd > 3 and PLI > 1.3 (displayed in orange to red), confirming pronounced contamination burden and clear spatial heterogeneity attributable to anthropogenic sources.

### 3.4. Evaluation of the Potential Ecological Risk from Toxic Metals

**Figure 4:**
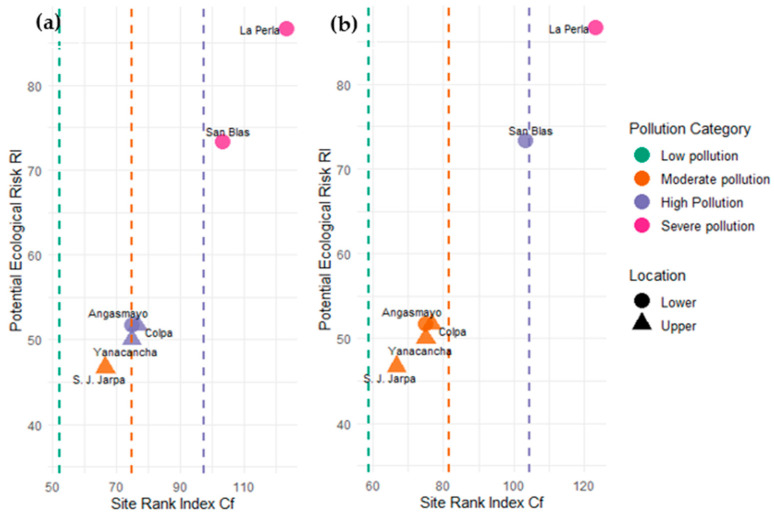
Behavior of the Site Ranking Index (SRI-Cf) in relation to the Potential Ecological Risk (RI) for heavy metals in sediments, according to the methods of (**a**) Taylor & Mclennan (1995) [26] and (**b**) Turekian & Wedepohl (1961) [27].

Figure 4 illustrates the relationship between Potential Ecological Risk Index (RI) and Site Rank Index (Cf) across sampling locations, with symbols differentiating upper zone sites (triangles) and lower zone sites (circles). The analysis employs two distinct geochemical normalization approaches: Panel (a) utilizes background values from Taylor & Mclennan (1995) [26], while Panel (b) applies reference concentrations from Turekian & Wedepohl (1961) [27]. Color coding represents pollution severity categories ranging from low (green) to severe (pink) contamination levels.

Both normalization methods demonstrate consistent spatial contamination patterns, with downstream sites exhibiting substantially higher ecological risk compared to upstream locations. La Perla consistently represents the most contaminated site across both panels, classified as severe pollution with RI values approaching 90 and Cf values exceeding 110–120. San Blas exhibits high pollution levels, with RI values ranging from 70 to 75 and Cf values between 90 and 100, depending on the background reference used. Angasmayo displays moderate to high pollution characteristics, positioned at intermediate risk levels between upstream sites and most downstream sites.

Upper zone sites (San Juan de Jarpa, Yanacancha, and Colpa) consistently cluster within the moderate pollution category, exhibiting RI values between 45 and 50 and Cf values ranging from 70 to 85. The tight clustering of these upstream sites suggests relatively uniform, lower-level contamination compared to the pronounced variability observed in downstream locations. The Taylor & McLennan normalization, Panel (a), generally yields slightly higher risk assessments compared to the Turekian & Wedepohl approach, Panel (b), reflecting differences in background reference concentrations between the two methodologies.

The vertical dashed lines delineate pollution category thresholds, facilitating rapid visual assessment of contamination severity. The clear spatial gradient from moderate pollution in upstream areas to severe pollution at downstream sites confirms progressive metal accumulation along the river continuum, consistent with point-source contamination from mining activities in the watershed. This pattern underscores the cumulative impact of anthropogenic inputs on sediment quality and ecological risk in the lower reaches of the Cunas River system.

### 3.5. Human Health Risk Assessment

The Chronic Daily Dose (CDD) values presented in Table A2 reveal greater exposure to heavy metals in the lower zone compared to the upper zone, particularly for As, Pb, Cr, and Cd. In locations such as San Blas and La Perla, the CDD for As ingestion reached levels of 2.6 × 10^−5^ mg/kg/day, while in the upper zone, these values were up to four times lower. Similarly, Pb in the lower zone showed CDD values > 3.5 × 10^−5^ mg/kg/day, significantly higher than those recorded in the upper zone (Table A3).

To assess non-carcinogenic and carcinogenic risks, the ingestion, dermal contact, and inhalation pathways were considered, in that order of importance. The results of the Hazard Quotient (HQ) indicated that ingestion is the primary risk pathway, with elevated values for As and Pb in the lower zone, exceeding the threshold of 1. This suggests a potential non-carcinogenic risk for exposed individuals. In contrast, in the upper zone, HQ values are lower and mostly below 1, indicating a reduced impact on human health.

**Figure 5:**
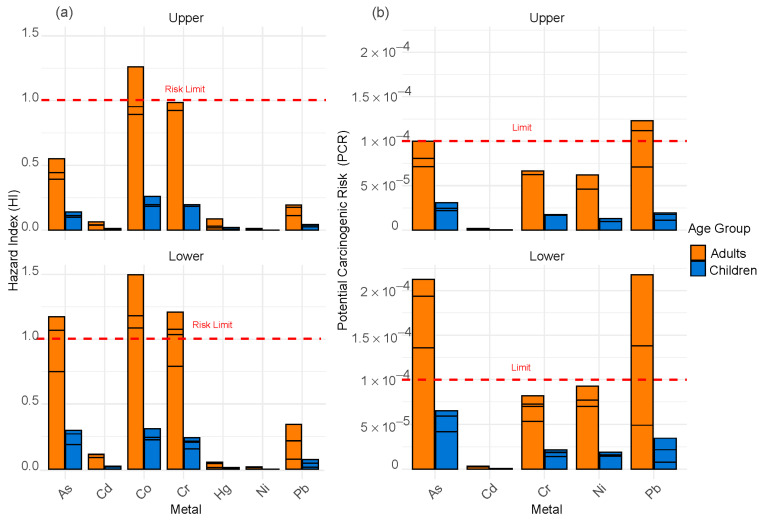
(**a**) Hazard Index (HI) and (**b**) Potential Carcinogenic Risk (PCR) for children and adults due to heavy metal exposure in sediments of the Cunas River.

Figure 5a shows the Hazard Index (HI) for adults and children exposed to heavy metals in the river sediments, distinguishing between the lower and upper zones. In the lower zone, Cd, Co, and As exhibit the highest HI values, exceeding the risk threshold in adults and approaching the limit in children. This indicates that exposure to these metals represents a significant health risk due to the greater accumulation of contaminants in the sediments. In the upper zone, although HI values are generally lower, Co and Cr exceed the risk threshold in adults, highlighting that these metals also pose a significant health threat in this region. Similarly, Figure 5b presents the Potential Carcinogenic Risk (PCR). In the lower zone, the highest PCR values are recorded for As and Pb, exceeding the risk threshold (1.0 × 10^−4^) in adults and approaching this limit in children. This suggests a greater long-term carcinogenic hazard in this area. A similar pattern is observed for Cr and Ni, though without reaching the critical threshold.

In the upper zone, PCR values are generally lower. However, As and Pb continue to stand out as the main contributors to risk, especially in adults, reflecting higher cumulative exposure compared to children. Although values in the child population are lower, metals such as As and Pb still show concerning levels, indicating that they should not be overlooked. These results demonstrate that the lower zone of the river is the most vulnerable to carcinogenic risks associated with heavy metal exposure, likely due to the greater accumulation of contaminants in the sediments in these areas.

### 3.6. Contribution of Metals to Carcinogenic Risk (HI and PCR) Across Zones and Age Groups

**Figure 6:**
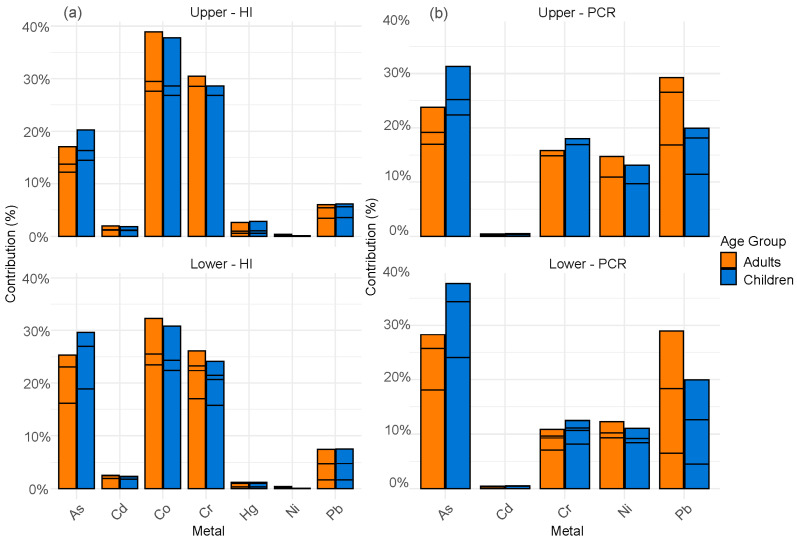
Contribution of metals to (**a**) Hazard Index (HI) and (**b**) Potential Carcinogenic Risk (PCR).

Figure 6 presents the contribution of metals in the upper and lower zones. In the lower zone, the metals with the highest contribution to HI are As, Cd, and Cr. As contributes up to 37% in adults and 33% in children, Cd accounts for 28% in adults and 24% in children, while Cr represents approximately 30% in adults and 27% in children, making them the primary contributors to non-carcinogenic risk in this area. Regarding PCR, the metals with the highest contribution are As, Cd, and Pb. As accounts for 42% in adults and 28% in children, Cd contributes approximately 16% in adults and 11% in children, while Pb represents 32% in adults and 19% in children. This suggests that exposure to these metals could be associated with an increased carcinogenic risk in this area.

In the upper zone, the metals with the highest contribution to HI remain As, Cd, and Cr. As contributes 26% in adults and 21% in children, while Cd accounts for 22% in adults and 17% in children. For Cr, the contribution reaches 24% in adults and 19% in children.

Regarding PCR, the metals with the highest contribution are As, Cd, and Pb. As shows a contribution of 36% in adults and 21% in children, Cd contributes 14% in adults and 9% in children, while Pb represents 28% in adults and 17% in children. Although the values are lower than in the lower zone, the pattern of higher exposure in adults remains, reaching up to 36% in some cases. However, children continue to be highly vulnerable to the toxic effects of these metals.

## 4. Discussion

### 4.1. Contamination Patterns and Ecological Risk

Heavy metal contamination in the Peruvian Central Highlands represents a critical environmental issue, with concentrations frequently exceeding environmental quality thresholds by up to ten times [45,46,47]. The pronounced downstream accumulation pattern observed in the Cunas River reflects the cumulative impact of mining activities and demonstrates how anthropogenic inputs progressively deteriorate aquatic ecosystems along the river continuum. This spatial contamination gradient is characteristic of mining-influenced watersheds globally, where point-source pollution from extractive activities creates distinct zones of environmental impact.

The contamination patterns documented in this study align with global trends in mining-impacted river systems. Similar spatial differentiation has been documented in the Yangtze River, where mining and industrial emissions led to downstream contamination [48], and in the Dianchi River, where persistent metal inputs drive ecological risk [49].

However, the severity of contamination in the Cunas River appears more pronounced than many comparable systems, with multiple metals simultaneously exceeding critical thresholds. This multi-metal contamination scenario contrasts with agriculture-dominated systems like the Wanchuan River, where specific elements dominate risk profiles [50], highlighting mining as the primary contamination source in the Cunas watershed.

The co-occurrence patterns of As, Cd, and Pb observed in downstream sediments suggest common geochemical sources and transport mechanisms, likely associated with polymetallic mining operations and inadequate tailing management. These elemental associations are particularly concerning from an ecological perspective, as they indicate simultaneous exposure to multiple toxicants with potentially synergistic effects. The bioavailability of metals in downstream sediments represents a critical factor determining ecological impact, with conditions favoring increased mobility and toxicity for aquatic organisms, similar to patterns documented in the Bakkhali River [51] and Yamuna River [52].

Seasonal dynamics, as reported by Moreno-Aguirre et al. (2024) [53] in Andean rivers, where runoff intensifies metal transport during rainy seasons, likely amplify contamination patterns in the Cunas watershed. This temporal variability suggests that metal loading may fluctuate significantly throughout the year, with peak contamination periods corresponding to increased hydrological activity. Understanding these seasonal patterns is crucial for developing effective monitoring and management strategies, as exposure risks may vary substantially between dry and wet seasons.

The methodological comparison between geochemical normalization approaches revealed important insights for contamination assessment in mining regions. The Taylor & Mclennan (1995) [26] framework demonstrated greater sensitivity to anthropogenic enrichment compared to that of Turekian & Wedepohl (1961) [27], suggesting its enhanced suitability for detecting mining-related contamination. This methodological sensitivity is particularly relevant for regulatory applications and risk assessment protocols in mining-influenced environments.

### 4.2. Human Health Risk Implications and Food Web Concerns

The human health risks identified in this study parallel concerning trends documented globally in mining-impacted river systems, establishing the Cunas River as a significant public health concern. The dominance of ingestion as the primary exposure pathway aligns with findings from the Swat River, Pakistan [54], and Yamuna River, India [52], where As and Pb exposure has been linked to serious health outcomes including cancer and developmental toxicity. This consistency across geographically diverse mining regions suggests common exposure mechanisms and reinforces the global significance of sediment-mediated health risks.

Carcinogenic risk patterns documented in the Cunas River are comparable to those reported in the Payra River, Bangladesh, and Nigerian river systems, where As, Cd, and Hg contribute significantly to lifetime cancer risk [55,56]. Similar threshold exceedances have been documented in Ethiopia [57], confirming a global pattern of health risks in mining-affected watersheds. The consistency of these patterns across diverse geographic and socioeconomic contexts underscores the universal nature of mining-related health threats and the need for standardized risk assessment approaches.

The proportional risk contributions identified are consistent with international assessments by Wu et al. (2023) [58], who identified Cd as the most enriched metal in fluvial sediments globally, and Hefmi et al. (2025) [59], who reported dangerous levels of Ni and Cd in fish tissues, demonstrating direct links between sediment contamination and bioaccumulation in crops and aquatic organisms. This represents a particularly critical concern in the Cunas watershed, where rural communities maintain high dependence on local food sources [60,61]. This dependency creates multiple exposure pathways that may compound health risks, particularly for vulnerable populations including children and pregnant women. The potential for biomagnification through aquatic food webs further elevates concerns, as predatory fish species may concentrate metals to levels far exceeding sediment concentrations.

### 4.3. Management and Research Implications

The severity of contamination documented in this study necessitates immediate implementation of comprehensive management strategies that address both source control and exposure reduction. The integration of advanced analytical approaches, including the spatial modeling techniques demonstrated by Saha et al. (2024) [62] in Mexico, could enhance contamination hotspot identification and guide targeted mitigation strategies.

The assessment of combined pollutant effects, including cyanide and mercury, as emphasized by Gwira et al. (2024) [63], represents a critical research priority for mining-influenced systems like the Cunas watershed. Current single-metal risk assessments may substantially underestimate actual risks when multiple contaminants act synergistically. Understanding these interactions is essential for developing realistic risk models and effective remediation strategies.

Current assessment limitations include temporal snapshot constraints and potential seasonal variability in metal mobility, particularly considering the transport dynamics identified by Moreno-Aguirre et al. (2024) [53].

The spatial variations in metal concentrations between sampling stations may be attributed to hydrological dynamics characteristic of the rainy season, particularly during February when precipitation reaches its peak intensity. During this period, intense precipitation events and high discharge pulses promote the transport, deposition, and remobilization of contaminated sediments along the river channel, resulting in localized concentration increases without altering the overall contamination trend. Studies in the Andean basins of southern Peru have documented seasonal increases in Al, Cd, Cu, Fe, Mn, Ni, and Pb concentrations during the wet season [15,64]. Similarly, fluvial sediments from tropical regions have shown elevated concentrations of Pb, Zn, Cu, and As during the rainy season relative to the dry season [65].

Future research priorities should include comprehensive temporal monitoring to capture seasonal variations, bioaccumulation assessments across complete food webs, spatial contamination modeling to predict transport patterns, and detailed evaluation of synergistic effects among multiple contaminants.

Effective long-term management requires integration of multiple approaches including source control through improved mining practices, implementation of remediation technologies such as phytoremediation and chemical stabilization, establishment of comprehensive monitoring networks, and active community engagement in watershed management. The development of region-specific sediment quality criteria and implementation of early warning systems would support regulatory enforcement and protect public health. Ultimately, sustainable solutions must address both immediate contamination threats and underlying governance issues that permit continued environmental degradation.

## 5. Conclusions

The present study revealed that heavy metal contamination in the sediments of the Cunas River represents a significant environmental and health risk. High concentrations of arsenic (As), lead (Pb), cadmium (Cd), zinc (Zn), and antimony (Sb) were identified, with values exceeding upper continental crust (UCC) reference levels, particularly in the lower watershed. This indicates heightened contaminant mobility and accumulation, increasing toxicity and bioaccumulation potential in aquatic life.

Ecological risk analysis identified La Perla and San Blas in the lower zone as hotspots of contamination and risk, directly linked to mining activities. Multiple indices confirmed severe ecological risk levels, demonstrating a clear spatial gradient from upstream to downstream areas. The Taylor & McLennan (1995) reference values provided greater sensitivity to anthropogenic enrichment compared to other methodologies.

Health risk assessments revealed that adults face greater cumulative metal exposure, with Hazard Quotients exceeding unity for As and Pb in the lower zone. Children remain highly susceptible, particularly to cadmium and arsenic, with carcinogenic risks approaching critical thresholds. The lower zone exhibits disproportionately higher risk contributions from these metals, demanding targeted mitigation.

To address these environmental and health concerns, we must implement continuous monitoring, remediation technologies such as phytoremediation, and improved mining practices. These integrated strategies are essential for reducing heavy metal contamination in the Cunas River watershed and safeguarding community health.

## Figures and Tables

**Table 1:** Descriptive statistics of heavy metals in river sediments (mg/kg) and sediment quality guidelines (UCC, TEL, PEL).

Type	Element	DS	Upper	Lower	UCC	TEL	PEL
Transition metals	Cu	Mean	6.2	8.1	45	18.7	108
		SD	2.2	2.6			
		Max	10.7	12.8			
	Cr	Mean	7.8	9.5	90	52.3	160
		SD	2.6	3.1			
		Max	13.2	13.2			
	Fe	Mean	9094	9986	47,200	-	-
		SD	1939	1607			
		Max	12,192	12,192			
	Mn	Mean	316	413	850	-	-
		SD	140	197			
		Max	531	752			
	Mo	Mean	0.5	0.9	2.6	-	-
		SD	0.2	0.4			
		Max	0.9	1.5			
	Ni	Mean	7.1	11	68	-	-
		SD	4.3	4.2			
		Max	16.4	16.4			
	V	Mean	23.4	26.9	130	-	-
		SD	4.5	2			
Post-transition metals	Cd	Mean	0.2	0.4	0.1	-	-
		SD	0.2	0.1			
		Max	0.5	0.5			
	Hg	Mean	0.2	0.1	0.4	0.13	0.7
		SD	0.1	0.1			
		Max	0.2	0.2			
	Pb	Mean	11.1	23.1	20	30.2	112
		SD	9.2	10.4			
		Max	31.8	31.8			
		Mean	35.9	101	95	124	271
	Zn	SD	48.9	58.7			
		Max	156	156			
		Mean	0.4	1.1	0.2	-	-
Metalloids	Sb	SD	0.2	0.8			
		Max	1.3	2.7			
		Mean	7.7	15.4	1.5	-	-
	As	SD	7.6	7.1			
		Max	23.9	23.9			

DS = (descriptive statistics); UCC = (upper continental crust); TEL = (Threshold Effect Level); PEL = (Probable Effect Level).

## Data Availability

The data are available by contacting corresponding authors for collaboration or other reasonable requests.

## Appendix A

**Table A1:** Contamination indices applied in the assessment of heavy metals in sediments.

Index	Equation	Contamination Categories	Reference
Cf	$CF=\frac{C_{m}\mathrm{sample}}{C_{m}\mathrm{background}}$	Low (<1), Moderate (1–3), High (3–6), Very High (≥6)	[29]
Igeo	$Igeo={log}_{2}\frac{C_{n}}{1.5\times B_{n}}$	Uncontaminated (<0), Low (0–1), Moderate (1–2), Moderate to High (2–3), High (3–4), High to Extreme (4–5), Extreme (>5)	[33]
PLI	$PLI=(Cf1\times Cf2\times Cf3\times Cfn{)}^{\frac{1}{n}}$	0 (Ideal ecosystem), 1 (Basic contamination levels), >1 (Progressive deterioration)	[31]
mCd	$mCd=\frac{\sum_{i=n}^{i=1}C_{f}^{i}}{n}$	Uncontaminated (≤1.5), Low (1.5–2), Moderate (2–4), High (4–8), Very High (8–16), Extremely High (16–32), Ultra High (>32)	[15]

Contamination Factor (Cf), Geoaccumulation Index (Igeo), Pollution Load Index (PLI), and Modified Contamination Degree (mCd).

**Table A2:** Significance relationship of heavy metals in river sediment (mg/kg).

Characteristic	Lower, N = 6 ^1^	Upper, N = 6 ^1^	*p*-Value ^2^
Al	5573.8333 ± 1730.4762	4710.3333 ± 1938.3396	0.5
Sb	1.1284 ± 0.7923	0.4082 ± 0.1191	0.004
As	15.4237 ± 7.1133	7.6582 ± 2.2380	0.065
Ba	88.2800 ± 26.7440	110.6100 ± 79.7292	>0.9
Be	0.2939 ± 0.0558	0.2443 ± 0.0721	0.2
Bo	5.9485 ± 2.2898	7.2113 ± 2.4421	0.4
Cd	0.4054 ± 0.1258	0.1889 ± 0.0501	0.009
Ca	109,415.0000 ± 19,860.5102	117,123.5000 ± 27,134.7066	0.8
Co	3.9005 ± 0.9129	3.0567 ± 0.8640	0.2
Cu	8.0985 ± 2.5829	6.1958 ± 2.6516	0.13
Cr	9.4828 ± 3.0827	7.8412 ± 2.6078	0.2
Sn	0.7381 ± 0.5195	2.1044 ± 3.3719	>0.9
Sr	136.5000 ± 14.2346	141.3433 ± 46.7255	0.7
Fe	9986.1667 ± 1607.0001	9094.0000 ± 2187.4855	0.3
Li	6.3513 ± 2.2018	6.8760 ± 3.5481	>0.9
Mg	8177.1667 ± 4729.7011	4261.5000 ± 953.6894	0.002
Mn	413.9333 ± 197.2829	316.1833 ± 138.9736	0.6
Hg	0.1090 ± 0.0563	0.1606 ± 0.0845	0.2
Mo	0.9428 ± 0.3608	0.5379 ± 0.1458	0.026
Ni	10.9673 ± 4.1551	7.1133 ± 2.2742	0.093
Ag	0.2209 ± 0.1789	0.0380 ± 0.0379	0.011
Pb	23.0670 ± 10.4145	11.1030 ± 4.8790	0.092
K	799.1667 ± 234.6085	738.8333 ± 273.1772	0.6
Se	0.5828 ± 0.1679	0.5095 ± 0.0862	0.3
SI	734.7833 ± 643.6402	993.0833 ± 1259.5507	0.8
Na	310.1167 ± 182.9327	287.0833 ± 166.8301	0.8
Tl	0.1992 ± 0.0824	0.1541 ± 0.0792	0.13
Ti	164.5000 ± 30.1517	175.9500 ± 76.4781	>0.9
Va	26.9467 ± 1.9740	23.3617 ± 5.1338	0.4
Zn	101.2833 ± 58.7266	35.8633 ± 6.5779	0.013

^1^ Mean ± SD; ^2^ Wilcoxon rank sum exact test.

**Table A3:** Contamination Factor, Pollution Load Index, and Modified Contamination Degree of heavy metals in sediments of the Cunas River.

		Contamination Factor—Cf														PLI	mCD
Site		Cu	Cr	Fe	Mn	Hg	Mo	Ni	Pb	V	Zn	Sb	As	Cd	Mn		
Yanacancha	Upper	0.10	0.06	0.17	0.26	0.29	0.17	0.09	0.37	0.14	0.34	3.64	3.64	1.11	0.29	0.78	0.10
S. J. Jarpa	Upper	0.09	0.06	0.15	0.25	0.53	0.28	0.07	0.34	0.13	0.39	4.13	4.13	1.06	0.34	0.85	0.09
Colpa	Upper	0.24	0.12	0.26	0.62	0.40	0.18	0.16	0.39	0.22	0.39	7.51	7.51	1.13	0.50	1.44	0.24
San Blas	Lower	0.18	0.15	0.26	0.55	0.29	0.33	0.24	1.58	0.18	0.63	15.96	15.96	3.50	0.72	3.04	0.18
Angasmayo	Lower	0.14	0.06	0.19	0.28	0.47	0.29	0.10	1.59	0.22	1.62	4.63	4.63	1.78	0.50	1.19	0.14
La Perla	Lower	0.28	0.13	0.23	0.46	0.23	0.56	0.16	1.59	0.22	1.62	11.47	11.47	3.18	0.74	2.41	0.28
Yanacancha	Upper	0.09	0.06	0.14	0.53	0.75	0.27	0.07	0.90	0.17	0.48	3.95	3.95	1.94	0.42	0.97	0.09
S. J. Jarpa	Upper	0.14	0.11	0.22	0.31	0.28	0.16	0.11	0.82	0.22	0.39	6.04	6.04	1.20	0.42	1.21	0.14
Colpa	Upper	0.17	0.11	0.22	0.26	0.16	0.18	0.12	0.52	0.20	0.27	5.36	5.36	1.12	0.38	1.07	0.17
San Blas	Lower	0.18	0.13	0.24	0.51	0.13	0.24	0.20	0.79	0.21	1.64	14.53	14.53	3.46	0.64	2.82	0.18
Angasmayo	Lower	0.12	0.07	0.19	0.24	0.39	0.24	0.08	1.01	0.20	0.41	4.92	4.92	1.59	0.41	1.08	0.12
La Perla	Lower	0.18	0.10	0.17	0.88	0.12	0.51	0.18	0.36	0.21	0.48	10.19	10.19	2.70	0.54	2.01	0.18

Contamination Factor (Cf), Pollution Load Index (PLI), and Modified Contamination Degree (mCd) of heavy metals in sediments of the river under mining influence, according to the sampling location based on Turekian & Wedepohl (1961).
